# Exploring the Impact of Coconut Peat and Vermiculite on the Rhizosphere Microbiome of Pre-Basic Seed Potatoes under Soilless Cultivation Conditions

**DOI:** 10.3390/microorganisms12030584

**Published:** 2024-03-14

**Authors:** Kan Yan, Yanni Ma, Songming Bao, Wandi Li, Yunjiao Wang, Chao Sun, Xin Lu, Juan Ran

**Affiliations:** 1 School of Biological and Pharmaceutical Engineering, Lanzhou Jiaotong University, Lanzhou 730070, China; shiyiadamyn@163.com (Y.M.); baosm0819@163.com (S.B.); liwandi2022@163.com (W.L.); lu_xin2022@163.com (X.L.); ranjuan0415@163.com (J.R.); 2 College of Agronomy, Gansu Agricultural University, Lanzhou 730070, China; wangyunjiao0924@163.com (Y.W.); sunc@gsau.edu.cn (C.S.)

**Keywords:** coconut peat, vermiculite, microbial diversity, community function, Potato Breeding

## Abstract

Soilless cultivation of potatoes often utilizes organic coconut peat and inorganic vermiculite as growing substrates. The unique microbial communities and physicochemical characteristics inherent to each substrate significantly influence the microecological environment crucial for potato growth and breeding. This study analyzed environmental factors within each substrate and employed Illumina sequencing alongside bioinformatics tools to examine microbial community structures, their correlation with environmental factors, core microbial functions, and the dynamics of microbial networks across various samples. These included pure coconut peat (CP1) and pure vermiculite (V1), substrates mixed with organic fertilizer for three days (CP2 and V2), and three combinations cultivated with potatoes for 50 days (CP3, V3, and CV3—a 1:1 mix of coconut peat and vermiculite with organic fertilizer). Vermiculite naturally hosts a more diverse microbial community. After mixing with fertilizer and composting for 3 days, and 50 days of potato cultivation, fungal diversity decreased in both substrates. Coconut peat maintains higher bacterial diversity and richness compared to vermiculite, harboring more beneficial bacteria and fungi, resulting in a more complex microbial network. However, vermiculite shows lower bacterial diversity and richness, with an accumulation of pathogenic microorganisms. Among the 11 environmental factors tested, water-soluble nitrogen (WSN), total nitrogen (TN), available potassium (AK), total organic carbon (TOC) and air-filled porosity (AFP) were significantly associated with microbial succession in the substrate.The nutritional type composition and interaction patterns of indigenous microorganisms differ between vermiculite and coconut peat. Adding abundant nutrients significantly affects the stability and interaction of the entire microbial community, even post-potato cultivation. When using vermiculite for soilless cultivation, precise control and adjustment of nutrient addition quantity and frequency are essential.

## 1. Introduction

The potato is globally recognized as the fourth largest food crop [1]. Due to its affordability, minimal cultivation requirements, and rich nutritional profile, it holds a significant position in the world’s agricultural market [2,3,4,5]. To preserve the taste, nutritional value, and economic importance of various potato strains, producing healthy and genetically stable seed potatoes, including pre-basic seed or foundation seed, is crucial for commercial production.

Soilless cultivation systems, as opposed to traditional field planting and pure culture medium cultivation, offer advantages such as enhanced water and nutrient use efficiency, simplified management, and the ability to address challenges including slow plant regeneration, medium browning, abnormal morphology, and regressive growth [6]. Consequently, seed breeding methods that incorporate aeroponics cultivation and substrate cultivation within greenhouses have become extensively adopted in commercial settings.

Coconut peat and vermiculite are key natural substrates in the organic and inorganic soilless seedling industries, respectively [7,8]. Coconut peat, a byproduct of the *Cocos nucifera* industry, constitutes about 25% of total coconut production and mainly consists of short fibers and coconut peat dust [9], along with high salt levels [10]. Its excellent rewettability and optimal air-water balance are beneficial for plant growth, although its low pH and limited cation exchange capacity necessitate fertilization to address these chemical shortfalls [11,12]. Vermiculite, a phyllosilicate mineral formed from the hydrothermal alteration of mica minerals such as biotite and phlogopite, is lightweight and resistant to heat decomposition [13]. It also has a lower bulk density, conductivity, and specific surface area, but higher total porosity and cation exchange capacity, richer in calcium and sulfur, and demonstrates greater adsorption capacities for NH_4_^+^, NO_3_^−^, PO_4_^3−^, and K^+^ [14].

The rhizosphere, the zone surrounding and directly in contact with plant roots, is a dynamic environment where the exchange of various environmental factors and microbial communities occurs. Plant secretions of sugars, amino acids, and other substances into the rhizosphere attract microbial colonization, facilitating nutrient exchange, availability, and sequestration by linking plants with soil microbes and environmental factors [15]. Beneficial rhizosphere microbes can protect plants from pathogenic infections through competitive resource utilization, antibiotic production, and plant growth promotion via substances such as IAA. They also transform recalcitrant substances into forms more accessible for plant use. However, the presence of pathogens within this complex microbial ecosystem can predispose plants to diseases and mortality through effector molecule secretion, direct parasitism, and other mechanisms, highlighting the sensitivity of plant growth and development to rhizosphere conditions.

While considerable research has been conducted on microbial communities in soilless cultivation systems, the focus has predominantly been on hydroponic systems, with substrate cultivation receiving comparatively less attention. It has been established that the choice of substrate plays a critical role in shaping the rhizosphere microbial community [16]. Oliver G. et al. [17] observed in their study on eggplant cultivation that a mix of coconut peat and peat substrates significantly enriched species from the *Proteobacteria* and *Actinobacteria* families, such as *Rhodocyclaceae* and *Methylophilaceae*, whereas *Chitinophagaceae* were more prevalent in rockwool substrates. Coconut peat, specifically, has been recognized for harboring beneficial fungi and plant growth-promoting rhizobacteria (PGPR) such as *Trichoderma* spp., *Paecilomyces fumosoroseus*, and *Galactomyces geotrichum*, which contribute to soilborne pathogen resistance, abiotic stress tolerance, and plant growth enhancement [18,19,20,21].

In contrast, research on the microbial community within vermiculite substrates, or even on pure vermiculite surfaces, remains scarce. Investigations into phyllosilicate mineral, from which vermiculite originates, and materials with similar structures to vermiculite, indicate that surface microorganisms primarily belong to two groups: those involved in rock weathering and organotrophic microbes [22]. These microbial populations are generally low in abundance and often thermotolerant, with many exhibiting a mix of biological traits [23,24]. Yet, their ecological roles are still not fully understood. Unique environmental pressures facilitate complex interactions between these microbial groups, enabling efficient nutrient utilization from challenging sources. This involves rock weathering microbes aiding the organic nutrient process through mechanisms such as organic acid hydrolysis and chelation, alongside the transformation and release of redox-active elements (e.g., Fe, Na), which in turn, enhance siderophore biosynthesis pathways and cation dissolution capacity [24,25,26,27]. Consequently, the nutritional environment surrounding mica minerals influences microbial composition, with reducing bacteria and fungi such as *Aspergillus*, *Cladosporium*, and *Penicillium* being prevalent. Additionally, the incorporation of organic media can lead to the enrichment of PGPR, including *Pseudomonas* spp. [28,29].

Although soilless cultivation initially aimed to shield plants from soilborne pathogens, contamination through plant materials, growth media, water sources, and other means is still a significant concern [16]. It is important to acknowledge that the long-standing adaptive relationship between plants and soil microenvironments results in a relatively stable indigenous microbial community, essential for disease suppression and plant growth promotion. In the absence of these indigenous soil microorganisms, soilless cultivation systems may experience more frequent and severe disease outbreaks. For instance, novel *Agrobacterium* species have caused root mat outbreaks affecting 30% of hydroponically grown tomatoes [30]. *Pythium* dissotocum has led to a 60% mortality rate in hydroponic spinach and root rot in lettuce [31,32]. Similarly, substrate cultivation has faced challenges with pathogens such as *Multinucleate Rhizoctonia Solani AG4 HG-1*, *Pythium myriotylum*, and *Diaporthe eres*, causing significant damage to peat-based crops such as strawberries and medical cannabis, with infection or mortality rates ranging from 20% to 35% [33,34,35].

In summary, the rhizosphere’s microbial milieu is pivotal to plant growth and development, yet research into microbial communities within soilless cultivation substrates, especially vermiculite, remains inadequate [36]. The scarcity of organic nutrients in such substrates limits plant growth and the colonization of roots by potential biocontrol agents, necessitating the addition of external fertilizers for nutrient adjustment. During this phase, efficient composting accelerates the breakdown of macromolecules in fertilizers, while the heat and nutrients produced by fermentation modify the microbial composition, encouraging the proliferation of both beneficial and detrimental microbes. Upon planting, there is a notable increase in the diversity and abundance of microbial communities [37]. Characterizing the microbial community at this stage is vital for elucidating the microbial dynamics throughout the cultivation process. Most existing studies neglect this critical phase. Our research aims to bridge this gap by analyzing the microbial diversity at three distinct stages: the initial substrate, post-composting at three days, and the rhizosphere substrate after 50 days of potato cultivation. This investigation seeks to uncover the direct and/or potential effects of coconut peat and vermiculite on potato seed breeding from a microbial standpoint, offering insights into the observed phenomena and contributing to filling the existing gaps in research.

## 2. Materials and Methods

### 2.1. Soil Sample Collection

**Figure 1:**
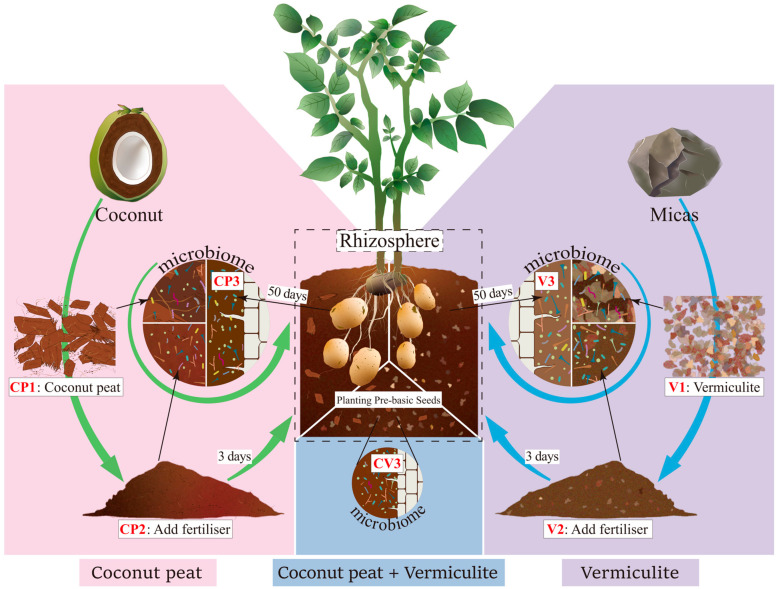
Sampling schematic. Samples were collected from two different substrates across three growth stages. Additionally, rhizosphere samples were obtained 50 days post potato planting in a coconut peat and vermiculite mix.

Samples were obtained from the soilless potato cultivation experimental greenhouse at Gansu Agricultural University. The sample processing flow is summarized in Figure 1. Initially, three substrate types were prepared: pure coconut peat (CP1), pure vermiculite (V1), a 1:1 mixture of coconut peat and vermiculite (CV1). These were stored without base fertilizer. Subsequently, a base fertilizer (15-15-15 compound fertilizer, contains 15% nitrogen, 15% phosphorus and 15% potassium) was added to each substrate and allowed to ferment outdoors for three days (everage daily temperature: 24 °C). Post-fermentation, samples were collected from these substrates: coconut peat with base fertilizer (CP2), vermiculite with base fertilizer (V2), a 1:1 mixture of coconut peat and vermiculite with base fertilizer (CV2). Healthy seed potatoes of the Feiwuruita variety, consistent in size, shape, and weight and free from surface diseases, were planted in these fermented substrates. The substrates were irrigated every five days. After 50 days, five potato plants from each substrate type were randomly selected, and samples from the substrate attached to their fibrous roots and tuber surface were collected, resulting in three groups of rhizosphere samples: CP3: planted potatoes in CP2 for 50 days, V3: planted potatoes in V2 for 50 days, CV3: planted potatoes in a 1:1 mix of coconut peat and vermiculite with organic fertilizer for 50 days. Samples from each period (CP1~CP3, V1~V3, CV3, totaling seven types) were mixed evenly and divided into three equal parts (each >100 g), representing three replicates. All samples were transported on dry ice and stored at −80 °C until further analysis.

### 2.2. Determination of Substrate Physicochemical Properties

Soil pH was measured using a pH meter after a 30-min soil-water immersion (vermiculite: water = 10:1, coconut peat: water = 20:1). Electrical conductivity (EC) was gauged at 25 ± 1 °C using a conductivity meter after preparing a leaching solution at 20 ± 1 °C (vermiculite: water = 10:1, coconut peat: water = 20:1). Total organism carbon (TOC) was assessed via the potassium dichromate oxidation-external heating method. Total nitrogen (TN) was determined through the Kjeldahl method. Total phosphorus (TP) was quantified using molybdenum antimony colorimetry following a strong alkali fusion of the sample. Total potassium (TK) was analyzed by flame photometry after alkali and dilute sulfuric acid fusion of the sample. Available phosphorus (AP) content was measured in the leaching solution (0.5 mol/L NaHCO3 extraction) using molybdenum antimony colorimetry. Available potassium (AK) content was determined in the leaching solution (1.0 mol/L ammonium acetate extraction) via flame photometry. Water-soluble nitrogen (WSN) was assessed by the alkaline hydrolysis diffusion method. Soil porosity (SP) was calculated as the weight difference between the water-soaked (24 h) and dry samples. Air-filled porosity (AFP) was the weight difference between the 24-h soaked sample and the sample post 3-h inversion post-soaking. Water-holding porosity (WHP) was the weight difference between the sample inverted and placed for 3 h after soaking and the dry sample before soaking.

### 2.3. Sample DNA Extraction, Amplification, and Sequencing

DNA extraction, amplification, and sequencing of the samples were outsourced to Biomarker Technologies Co., Ltd., Beijing, China. Total genomic DNA was extracted using the Tiangen Biotech (Beijing, China) Co., Ltd. DNA extraction kit (Model: DP812). Nucleic acid concentration was verified using an enzyme labeler (Gene Company Limited, Model: synergy HTX). The bacterial 16S rRNA gene was amplified with primers 338F (5′-ACTCCTACGGGAGGCAGCA-3′) and 806R (5′-GGACTACHVGGGTWTCTAAT-3′), and the fungal ITS1 gene region with primers ITS1F (5′-CTTGGTCATTTAGAGGAAGTAA-3′) and ITS2 (5′-GCTGCGTTCTTCATCGATGC-3′). Amplicons were purified using VAHTSTM DNA Clean Beads (Novgene Co., Ltd., Nanjing, China) and assessed via 1.8% agarose gel electrophoresis (Biomarker Technologies Co., Ltd., Beijing, China). Quantification was performed using ImageJ software (version 1.53s) to standardize the construction of the sequencing library. Sequencing was conducted on the Illumina Novaseq 6000 platform.

### 2.4. Bioinformatics Analysis

Raw readsobtained from sequencing were filtered using Trimmomatic (version 0.33) [38]. Primer sequences were recognized and removed using Cutadapt (version 1.9.1) [39], producing clean readsdevoid of primer sequences. Clean reads from each sample were assembled via overlap using Usearch (version 10) [40], followed by length filtering. Denoising and chimera sequence removal were performed using the dada2 [41] method in QIIME2 (version 2020.6) [42], yielding Non-chimeric reads. Each sample produced 45,054 to 65,138 bacterial Non-chimeric reads and 74,470 to 78,573 fungal Non-chimeric reads. Bacterial ASVs ranged from 448 to 1758, and fungal ASVs from 353 to 870. Bacterial and fungal ASVs were classified and annotated using the SILVA [43] and UNITE [44] databases, respectively, with a naive Bayes classifier. After the removal of Unassigned_Bacteria, the final sequences underwent alpha and beta diversity analyses, integrating environmental factors.

### 2.5. Statistical Analysis

The alpha diversity index of the samples was evaluated using QIIME2 software (version 2020.6) [42], with the t-test applied to assess differences in alpha diversity between treatments. Species distribution histograms and chord diagrams were created using the BMKCloud platform’s online analysis tool (http://www.biocloud.net, accessed on 5 August 2022) to visualize species composition and distribution. Non-metric multidimensional scaling (NMDS) analysis, based on the Bray-curtis distance matrix, was performed using QIIME software (version 1.9.1) [45] to examine bacterial and fungal community differences among groups. Canonical correspondence analysis (CCA) in Canoco5 [46] analyzed the relationships between the samples’ physicochemical properties, microbial community, and phylum-level microorganism composition, followed by constrained analysis to derive final results. Correlations between environmental factors and microorganisms were visualized using the “pheatmap” package [47] in R version 4.2.1 [48]. Linear discriminant analysis effect size (LEfSe) was conducted on the Galaxy platform [49] to identify specific microbial communities across groups. The “tidyverse” [50,51] and “ggtern” [52] packages in R processed and visualized ternary diagram enrichment data, respectively. Variance partitioning analysis (VPA), using the “vegan” package [53], was combined with CCA results to explore the relationship between community composition, dominant bacterial phyla, and environmental factors. The “psych” [54] and “pheatmap” packages further analyzed these correlations. Functional composition changes in the community were predicted using the FAPROTAX database (1.2.6) [55,56] (http://www.stbates.org/funguild_db.php, accessed on 8 August 2022) for bacteria and the FunGuild database (1.0) [57,58] for fungal trophic composition. Bacterial metabolic pathway changes were projected using the KEGG database, provided by BMKCloud (http://www.biocloud.net, accessed on 5 August 2022.). Co-occurrence network analysis, based on the Spearman test with BH-corrected *p* values, was performed using the “igraph” [59] and “Hmisc” [60] packages in R, and network correlations were visualized using Gephi software (0.9.7).

## 3. Results

### 3.1. Changes in Microbial Community Structure

The flattening of the rarefaction curve (Figure S1) suggests that the sequencing depth adequately captured the primary composition of the microbial community in the sample.

#### 3.1.1. Changes in Bacterial Community Diversity and Richness

Bacterial community richness and diversity were assessed for each treatment group (Table S1a). Across the three stages of different substrate treatments, Alpha diversity indices exhibited similar trends. In the CP1~3 series, with coconut peat as the substrate, both Shannon and Simpson indices initially decreased and then increased from CP1 to CP3. However, the indices in CP3 remained lower than in CP1. The Chao1, ACE, and PD_whole_tree indices significantly decreased initially and then increased, but still, the values for CP3 were lower than those for CP1. In the V1~3 series, using vermiculite as the substrate, a similar pattern was observed with the Shannon and Simpson indices decreasing first and then slightly increasing, though the final indices for V3 remained lower than for V1. The Chao1, ACE, and PD_whole_tree indices initially dropped sharply and then slightly increased.

Comparing the same treatments across different substrates, notable differences in Alpha diversity indices were observed. In the untreated pure substrates CP1 and V1, V1 exhibited a higher Shannon index, while the Simpson index showed no significant difference. The ACE and Chao1 indices were significantly higher in V1 than in CP1, but the PD_whole_tree index was lower. In CP2 and V2, which were treated with base fertilizer and fermented for 3 days, the patterns in Shannon, Simpson, and PD_whole_tree indices were similar to their respective CP2 and V2 values, but the ACE and Chao1 indices were significantly lower in V2 than in CP2. Furthermore, in the CP3, V3, CV3 groups, where potatoes were cultivated for 50 days, the higher the proportion of coconut peat, the greater the diversity (Shannon, Simpson) and richness (ACE, Chao1, PD_whole_tree) indices, with CP3 having the highest values followed by CV3 and V3.

#### 3.1.2. Changes in Fungal Community Diversity and Richness

Fungal community richness and diversity were assessed for each treatment group (Table S1b). In the coconut peat substrate, the CP2 and CP3 groups exhibited significantly lower diversity (Shannon, Simpson) and richness (ACE, Chao1, PD_whole_tree) indices compared to CP1, with no notable differences between CP2 and CP3. For the vermiculite substrate, groups V1 and V2 did not show significant changes in any diversity parameters. However, in group V3, post 50d-planting, the Shannon and Simpson indices were significantly reduced, while ACE, Chao1, and PD_whole_tree indices remained unchanged.

Contrasting with bacterial communities, 50 days post-planting, samples with higher coconut peat content (CP3) demonstrated the lowest values for all five indices (CP3 < V3/CV3). Regarding diversity, the mixed coconut peat and vermiculite samples (CV3) had the highest Shannon and Simpson indices (CV3 > V3 > CP3), with significant differences noted. In terms of richness, the vermiculite-containing substrates displayed significantly higher ACE, Chao1, and PD_whole_tree indices, increasing as the proportion of vermiculite rose (V3 > CV3 > CP3). However, PD_whole_tree indices did not significantly differ between V3 and CV3 (V3/CV3 > CP3).

#### 3.1.3. Changes in the Structure of Bacterial and Fungal Communities

1. ASV-level analyses showed that the community compositions under different treatments were significantly different.

Community compositions under different treatments varied significantly, as shown by NMDS analysis (Figure S2). This analysis indicated that the three biological replicates within the same treatment group clustered closely, reflecting high similarity in community structure. The Dry Vermiculite group (V1) exhibited lower similarity compared to other groups, while the fungal community composition similarities among groups were less pronounced.

2. Trends in community changes at the phylum level.

**Figure 2:**
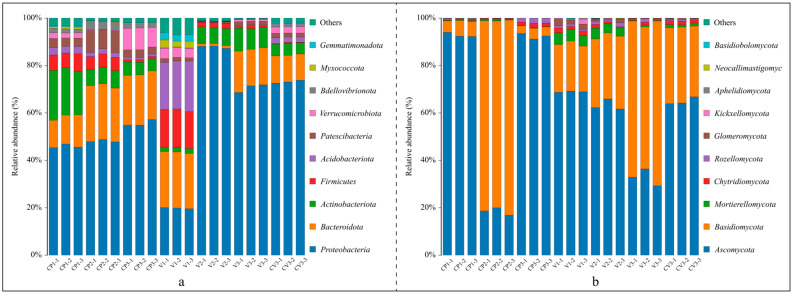
Bar graphs depicting the top ten phyla by relative abundance under various treatments: (**a**) Bacterial relative abundance; (**b**) Fungal relative abundance.

To elucidate microbial community changes within the same substrate and differences across substrates, we first referenced relevant databases to analyze bacterial and fungal phyla and genera in the samples. We identified 38 bacterial phyla and 15 fungal phyla. Figure 2 displays the top ten phyla in terms of relative abundance. The core bacterial phyla included Proteobacteria (19.8~87.8%), Bacteroidota (1.1~23.6%), Actinobacteriota (2.0~19.7%), Firmicutes (0.3~15.9%), Acidobacteriota (0.1~20.5%), Patescibacteria (0.5~8.8%), Verrucomicrobiota (0.1~8.9%), Bdellovibrionota (0.1~1.7%), Myxococcota (0.0~2.9%), and Gemmatimonadota (0.0~2.8%), collectively accounting for over 93% (93.3~99.7%) of the microbial composition in all treatments.

The bacterial community compositions at the phylum level varied across groups (Figure 2a). In the coconut peat treatments, relative to dry coconut peat, the relative abundances of *Bacteroidota*, *Patescibacteria*, and *Bdellovibrionota* increased after a 3-day base fertilizer application, while *Actinobacteriota*, *Verrucomicrobiota*, and *Firmicutes* decreased significantly. After 50 days of potato cultivation, there were no substantial changes in *Bacteroidota* content, but *Proteobacteria* and *Verrucomicrobiota* increased, and *Patescibacteria* and *Bdellovibrionota* returned to dry coconut peat levels. The relative abundance of *Acidobacteriota* and *Firmicutes* continued to decline in all three groups. In the vermiculite treatments, compared to dry vermiculite, *Proteobacteria* and *Actinobacteriota* increased significantly post 3d-fertilization, while *Bacteroidota*, *Firmicutes*, *Acidobacteriota*, *Patescibacteria*, *Verrucomicrobiota*, *Myxococcota*, and *Gemmatimonadota* saw substantial reductions. Fifty days after potato planting, *Bacteroidota*, *Actinobacteriota*, and *Patescibacteria* abundances significantly rose, whereas *Proteobacteria* and *Firmicutes* decreased. Overall, *Proteobacteria*, *Bacteroidota*, and *Actinobacteriota* were predominant in all groups. Notably, dry vermiculite initially exhibited greater species diversity than dry coconut peat, but this advantage diminished following fertilization and cultivation.

Fungal community compositions at the phylum level also varied (Figure 2b). In the coconut peat treatments, the dominant phyla were *Ascomycota*, *Basidiomycota*, *Chytridiomycota*, and *Rozellomycota*. Following the addition of base fertilizer and subsequent fermentation, *Ascomycota*’s relative abundance notably decreased while *Basidiomycota* increased. After 50 days of potato cultivation, *Ascomycota*’s abundance rebounded to the level of dry coconut peat, while *Basidiomycota* decreased substantially. The consistent presence of *Chytridiomycota* and *Rozellomycota* highlighted their adaptability in these samples.

In the vermiculite treatments, predominant phyla included *Ascomycota*, *Basidiomycota*, *Mortierellomycota*, *Chytridiomycota*, *Rozellomycota*, and *Glomeromycota*. Compared to dry vermiculite, the relative abundances of *Ascomycota*, *Mortierellomycota*, and *Chytridiomycota* decreased continually after base fertilizer application and potato planting, while *Basidiomycota* consistently increased. Post-experiment, *Ascomycota* and *Basidiomycota* were the most dominant in all three treatments, although the leading phylum varied between samples.

3. Community changes at the genus level.

**Figure 3:**
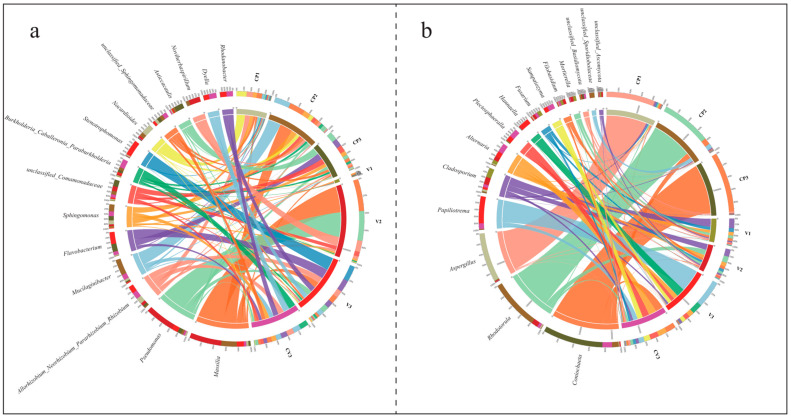
Distribution and chord diagrams illustrate the top 15 genera by relative abundance under various treatments. The left half of the diagram showcases the top 15 fungal genera in relative abundance across all samples, with different colors in adjacent curved columns representing different sample groups. A greater color proportion suggests a higher frequency of sequences from that genus in the respective group. The right half depicts different sample groups, where colors in adjacent curved columns denote different fungal genera. Here, a greater color proportion indicates a higher relative abundance of that genus within the group. The colors in the central band match the fungal genera’s colors, showing the association between genera and sample groups. The band’s thickness reflects relative abundance, with thicker bands denoting a higher relative abundance. (**a**) Bacteria; (**b**) Fungi.

In total, 1360 bacterial and 690 fungal genera were identified, comprising 71 core bacterial (5.2%) and 133 core fungal genera (19.3%). Significant variances in species composition and dominant genera were observed among groups (Figure S3). For example, the 15 most abundant bacterial genera displayed diverse environmental adaptations (Figure 3a, Table S2a). Notably, Massilia was prevalent in CP2 and V2, Pseudomonas in V2, Allorhizobium_Neorhizobium_Pararhizobium_Rhizobium in V3, CV3, and CP1, Mucilaginibacter in CP2, Flavobacterium in V3, CP3, CV3, and Sphingomonas in coconut peat environments (CP2, CV3, CP3, CP1). Burkholderia_Caballeronia_Paraburkholderia were mainly found in CV3, Stenotrophomonas almost exclusively in V3, Asticcaulis predominantly in CP3, and Noviherbaspirillum largely in V2. Dyella and Rhodanobacter were primarily present in CV3 and V3, which included diatomite. Furthermore, significant differences in dominant bacterial genera were noted across samples (Figure 3a, Table S2b). In groups CP1, CP2, and V2, the leading bacterial genera, in descending order, were Nocardioides, Allorhizobium_Neorhizobium_Pararhizobium_Rhizobium, Sphingomonas (CP1); Mucilaginibacter, Massilia, Sphingobacteriaceae (CP2); Massilia, Pseudomonas, Noviherbaspirillum (V2). The rhizosphere samples (CP3, V3, CV3) exhibited significantly higher uniformity in the distribution of dominant genera compared to other groups.

The principal fungal genera identified in the study, as shown in Figure 3b, are *Coniochaeta*, *Rhodotorula*, *Aspergillus*, *Papiliotrema*, *Cladosporium*, *Alternaria*, *Plectosphaeralla*, *Hannaella*, *Fusarium*, and *Sampaiozyma*. Their distribution and dominance varied markedly across different groups (Table S2c,d). *Coniochaeta* was primarily found in treatments containing coconut peat, particularly notable in CP3. *Rhodotorula* was predominantly observed in samples taken three days post-base fertilizer fermentation, especially in CP2. *Aspergillus* was almost exclusively identified in dry coconut peat (CP1). *Papiliotrema* mainly occurred in diatomite-inclusive rhizosphere samples (V3, CV3), with a significant presence in V3. Both *Sampaiozyma* and *Plectosphaeralla* showed adaptation to this specific environment. Additionally, *Hannaella* was primarily present in CV3, where it demonstrated a distinct advantage.

4. LEfSe analysis identified taxa contributing significantly to the community’s uniqueness in different groups.

**Figure 4:**
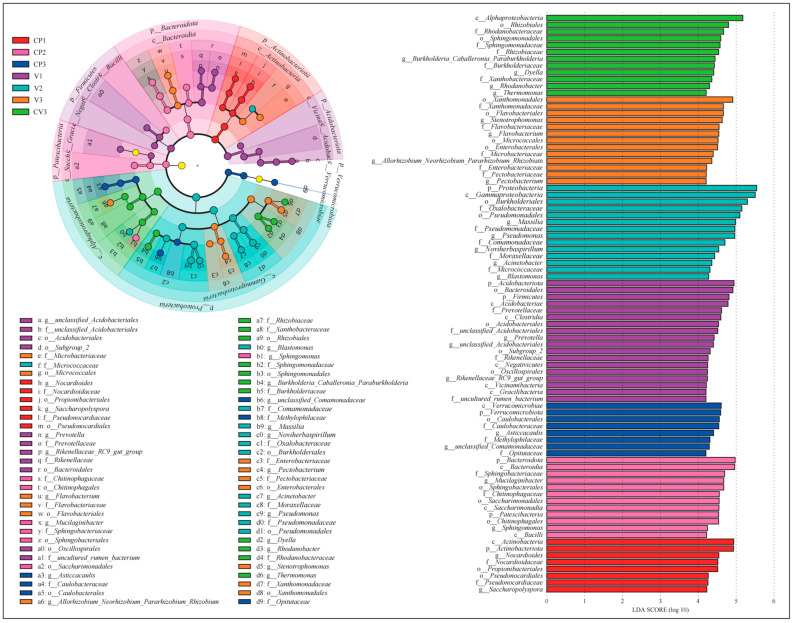
The linear discriminant analysis (LDA) effect size (LEfSe) analysis results for bacteria highlight taxonomic groups with statistical differences between the seven groups, ranging from phylum to genus (The non-parametric Kruskal-Wallis rank sum test is employed to analyze microbial populations exhibiting significant differences among multiple groups. Set LDA > 4.2). The left panel features an evolutionary branch diagram, where circles from the innermost to the outermost layer represent taxonomic levels from phylum to genus. Each small circle at various levels indicates a category at that taxonomic level, with the circle size reflecting its relative abundance. Circles colored correspondingly in the figure denote biomarkers of that treatment, while yellow circles represent categories without significant differences under the corresponding LDA values. The legend on the right details biomarkers at different taxonomic levels in each color group. The right panel is a bar chart displaying the distribution of LDA values, showing biomarkers in each group with an LDA score > 4.2.

For bacteria, the threshold was set at Linear Discriminant Analysis (LDA) score > 4.2, yielding 39 taxonomic groups (Figure 4, Table S3a). At the phylum level, Actinobacteriota was predominant in CP1, while Acidobacteriota and Firmicutes were prominent in V1 for the groups with a single type of dry substrate. At the genus level, Nocardioides and Saccharopolyspora were prevalent in CP1, while unclassified_Acidobacteriales, Prevotella, and Rikenellaceae_RC9_gut_group were prevalent in V1. In groups treated with high nutrient levels, Bacteroidota and Patescibacteria were abundant in CP2 at the phylum level, with Proteobacteria being abundant in V2. At the genus level, Mucilaginibacter and Sphingomonas were dominant in CP2, while Blastomonas, Acinetobacter, Noviherbaspirillum, Pseudomonas, and Massilia were dominant in V2. In all three rhizosphere soil groups, a substantial number of Proteobacteria (phylum) taxa were enriched, including Asticcacaulis (Caulobacterales class to genus), unclassified Comamonadaceae (genus), and Methylophilaceae (family) in CP3; Allorhizobium_Neorhizobium_Pararhizobium_Rhizobium (genus), Enterobacteriaceae (family under Enterobacterales order), Pectobacterium (Pectobacteriaceae family to genus), and Stenotrophomonas (Xanthomonadales order to genus) in V3; Rhizobiaceae and Xanthobacteraceae (Rhizobiales order to family), Sphingomonadaceae (Sphingomonadales order to family), and Burkholderia_Caballeronia_Paraburkholderia (Burkholderiaceae family to genus), Dyella and Rhodanobacter (Rhodanobacteraceae family to genus), Thermomonas (genus) in CV3. Additionally, CP3 was enriched with Opitutaceae (Verrucomicrobiae class to family) under Verrucomicrobiota (phylum), and V3 with Microbacteriaceae (Micrococcales order to family) and Flavobacterium (Flavobacteriales order to genus).

For fungi, the LDA threshold was set at >4.0, resulting in the identification of 22 taxonomic groups (Figure S4, Table S3b). Among the groups with dry substrates, *Ascomycota* was enriched in CP1 at the phylum level, while *Glomeromycota* and *Mortierellomycota* were prevalent in V1. At the genus level, *Aspergillus* was prominent in CP1, whereas *Mortierella*, *Fusarium*, and *Cladosporium* were prevalent in V1.

In the groups treated with high nutrient levels, *Basidiomycota* was the enriched phylum in CP2. At the genus level, *unclassified_Sporidiobolaceae* and *Rhodotorula* were prevalent in CP2, whereas *unclassified_Ascomycota*, *Fusicolla*, and *Filobasidium* were prevalent in V2. Among the three rhizosphere soil groups, *Coniochaeta* (classified from *Sordariomycetes* class to genus) was a biomarker in CP3; in V3, biomarkers included *Eotryotinia* (classified from *Helotiales* order to genus), *Hannaella*, and *Papiliotrema* under *Tremellomycetes* class; in CV3, biomarkers were *Alternaria* (classified from *Pleosporales* order to genus), *Plectosphaerella* (from *Glomerellales* order to genus), and *unclassified_Basidiomycota* (from order to genus).

### 3.2. Enrichment of Plant Growth-Promoting Rhizobacteria within Groups

**Figure 5:**
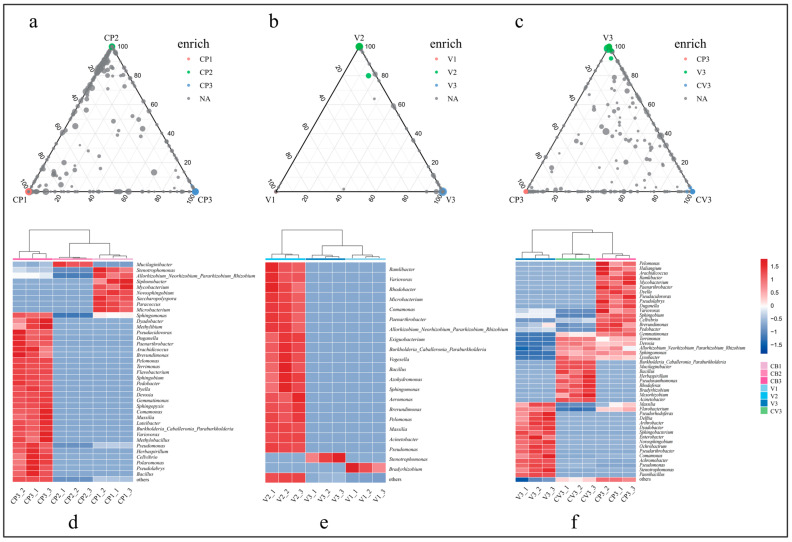
This figure illustrates the enrichment of bacterial ASVs and PGPRs between different treatments at a significance level of adjusted (BH) *p* < 0.01. Only ASVs with a relative abundance >0.1% were included. (**a**) ASV enrichment in treatments using coconut peat as the matrix; (**b**) ASV enrichment in treatments using vermiculite as the matrix; (**c**) ASV enrichment in three rhizosphere samples with different matrices; (**d**) Genus-level distribution of enriched ASVs in (**a**); (**e**) Genus-level distribution of enriched ASVs in (**b**); (**f**) Genus-level distribution of enriched ASVs in (**c**).

To analyze the enrichment characteristics of rhizosphere microorganisms, a ternary plot was utilized, visualizing the ASVs enriched across three treatments using different substrates (Figure 5, Table S4).

Based on literature review, enriched ASVs in different groups were categorized as PGPR at the genus level [61,62,63,64,65,66,67,68,69,70,71,72,73,74,75,76,77,78,79,80,81,82,83,84,85,86,87,88,89,90,91,92,93,94,95,96,97,98,99,100,101,102,103,104,105,106,107,108,109,110,111,112,113,114,115,116,117,118,119,120,121,122,123,124,125,126,127,128,129,130,131,132,133,134,135,136,137,138,139,140,141,142,143,144,145,146,147,148,149,150,151,152,153,154,155,156,157,158,159,160,161,162,163,164,165,166,167,168,169,170,171,172,173,174,175,176,177,178,179,180,181,182,183]. A total of 62 PGPRs were identified, exhibiting 11 plant growth-promoting functions such as phosphate solubilization, nitrogen fixation, IAA production, and siderophore production (Table S4a). In the coconut peat-based groups, 32 ASVs were enriched in CP1, 9 in CP2, and 276 in CP3’s rhizosphere soil, corresponding to 24, 5, and 139 genera, respectively (Figure 5a, Tables S4b and S5c). A heatmap visualized the distribution of 42 potential PGPRs among these groups (Figure 5d, Table S4c), revealing a notably higher count of PGPRs (33 types) in CP3 than in non-cultivated samples. Additionally, a considerable number of unclassified species enriched in CP3 have yet to be confirmed for plant growth-promoting functions.

In contrast, ASV enrichment in the vermiculite matrix (Figure 5b) showed a different pattern, with 4 ASVs enriched in V1, 72 in V2, and only 3 in V3’s rhizosphere soil. These ASVs were classified into 3, 25, and 3 genera, respectively, yielding 19 PGPRs (Tables S4d and S5e). The majority of these PGPRs were found in V2 (Figure 5e), possibly due to high concentrations of phosphorus, potassium, and nitrogen, as well as a large species base number.

Similarly, the enrichment analysis of rhizosphere soils with different inclusions revealed significant differences in the impact of substrate materials on microbial enrichment (Figure 5c). A total of 531 ASVs with significant differences were identified across three rhizosphere groups, with 253, 110, and 168 ASVs enriched in CP3, V3, and CV3, respectively, classified into 140, 59, and 91 genera (Table S4f). The categorization of their functions revealed that there are 28, 24, and 22 genera of PGPRs in the bacteria enriched in CP3, V3, and CV3, respectively (Figure 5f, Table S4g). Notably, numerous unclassified bacterial genera in CP3 and CV3 have yet to have their functions definitively determined.

### 3.3. Changes in Community Function and Metabolic Pathways

#### 3.3.1. Bacterial FAPROTAX Function Prediction

**Figure 6:**
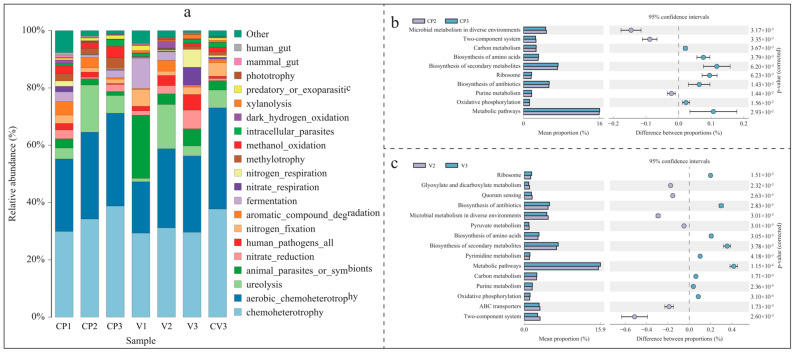
This figure illustrates the changes in bacterial community functions and metabolic pathways (species with relative abundance >1%, *p* < 0.05, a pairwise comparison between KEGG metabolic pathways in CP2 (or V2) and CP3 (or V3) was performed by applying t-test). (**a**) FAPROTAX environmental function analysis of bacterial communities under different treatments, highlighting the top 20 functional distributions by relative abundance. (**b**) Differential metabolic pathways in the coconut peat sample with base fertilizer added for 3 days (CP2) and the rhizosphere sample after 50 days of potato cultivation (CP3). (**c**) Differential metabolic pathways in the vermiculite sample with base fertilizer added for 3 days (V2) and the rhizosphere sample after 50 days of potato cultivation (V3).

Utilizing FAPROTAX for ecological function prediction of the identified bacteria (Figure 6a), the chart displays the top 20 species by relative abundance, representing 92.4% to 99.3% of each group’s total composition (Table S5a). All seven treatments exhibited a notably high abundance of chemoheterotrophy and aerobic chemoheterotrophy. The analysis indicates similarities in functional composition across different substrates at various stages. For instance, compared to single-substrate treatments of pure coconut peat (CP1) and pure vermiculite (V1), the introduction of base fertilizer (CP2, V2) increased the abundance of chemoheterotrophy, aerobic chemoheterotrophy, and ureolysis, while reducing the presence of animal parasites or symbionts and fermentation. Similarly, after 50 days of potato cultivation, both CP3 and V3 demonstrated a reduction in ureolysis and an increase in nitrate reduction. Pure vermiculite showed distinct functional compositions from other groups, particularly with high abundances of animal parasites or symbionts, nitrogen fixation, and fermentation. The functional composition in CV3 largely mirrored that of CP3, but with notable differences such as higher nitrogen fixation and lower fermentation, methylotrophy, and methanol oxidation, likely attributable to vermiculite’s specific characteristics.

#### 3.3.2. Bacterial KEGG Metabolic Pathway Changes

The Picrust2 analysis, based on the KEGG database, revealed differences in metabolic pathway expression within bacterial communities in the base fertilizer and rhizosphere samples (Figure 6b,c, Table S5b,c). Both coconut peat and vermiculite showed similar enrichments in rhizosphere metabolic pathways. Compared to the base fertilizer treatment groups (CP2, V2), the rhizosphere samples (CP3, V3) significantly enriched pathways in carbon metabolism, biosynthesis of amino acids, secondary metabolites, ribosome, antibiotics biosynthesis, oxidative phosphorylation, and general metabolic pathways (*p* < 0.05). Notably, in the vermiculite group, V3 also showed significant enrichment in pyrimidine and purine metabolism compared to V2.

#### 3.3.3. Changes in Fungal FunGuild Nutritional Composition

**Figure 7:**
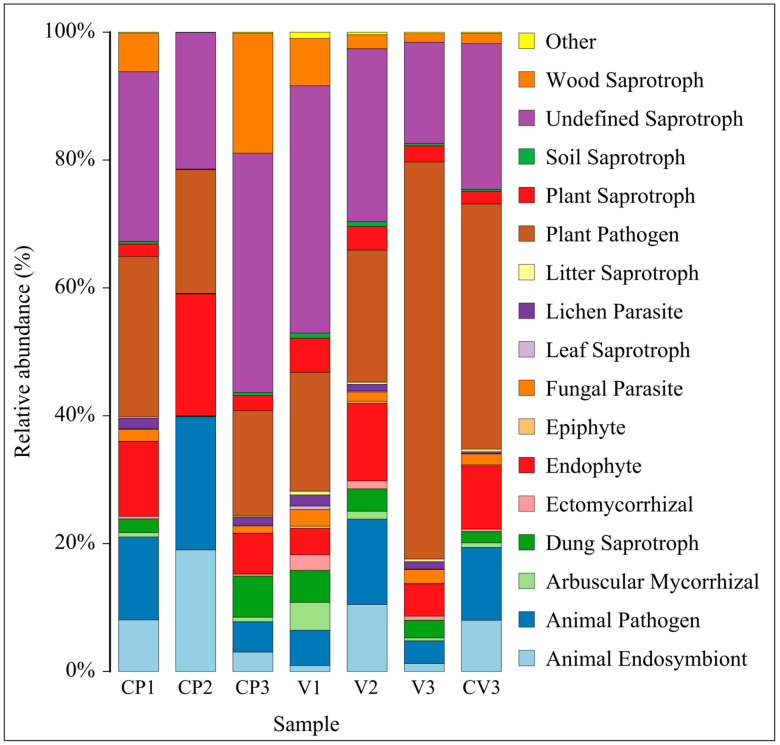
This figure presents an analysis of the fungal community’s nutritional types under different treatments using FunGuild. It displays the composition of nutritional types at the Guild functional level for species with a relative abundance greater than 0.1%.

Utilizing the FunGuild database, the analysis and visualization of fungal nutritional composition (relative abundance > 0.1%) under different environments were conducted (Figure 7), representing over 99% of the functional composition (Table S5d). In coconut peat, compared to CP1, the abundance of Animal Endosymbiont, Animal Pathogen, and Endophyte in CP2 (with added base fertilizer) increased, while the abundance of 13 other functions decreased, particularly Undefined Saprotroph and Wood Saprotroph. Except for Plant Pathogen, other nutritional types followed a similar trend in pure vermiculite (V1) and vermiculite with added base fertilizer (V2). In CP3, compared to CP2, the abundance of Dung Saprotroph, Undefined Saprotroph, and Wood Saprotroph (all Saprotrophs) increased, while the abundance of Animal Endosymbiont, Animal Pathogen, and Endophyte decreased, possibly due to continuous nutrient consumption. Differing from coconut peat, in the vermiculite-based groups, V3 did not show an increase in Dung Saprotroph, Undefined Saprotroph, and Wood Saprotroph compared to V2. Specifically, Undefined Saprotroph decreased significantly, and Plant Pathogen increased, which may be due to vermiculite’s lack of TOC and other physicochemical properties. Additionally, across the three rhizosphere samples, as the proportion of coconut peat increased and vermiculite decreased (V3→CV3→CP3), the relative abundance of Plant Pathogen decreased while Undefined Saprotroph increased.

### 3.4. Differences in Microbial Co-Occurrence Networks

**Figure 8:**
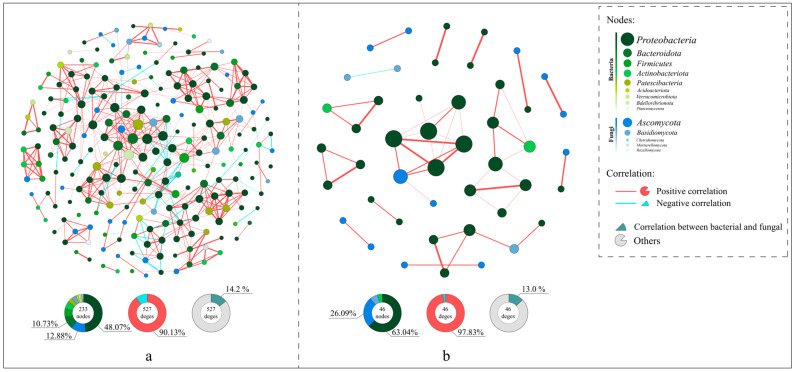
The co-occurrence network analysis and visualization include OTUs appearing in four or more groups, with Spearman’s coefficient (r) > 0.6 and an adjusted (BH) *p*-value < 0.01. In the network, green nodes represent bacteria, blue nodes represent fungi, and the size of each node indicates the relative abundance of the corresponding OTU. Red edges indicate positive correlations, blue edges indicate negative correlations, and edge thickness represents the strength of the correlation. (**a**) Co-occurrence network in the coconut peat substrate (CP1~CP3); (**b**) Co-occurrence network in the vermiculite substrate (V1~V3).

Spearman’s analysis was employed to explore the correlations between species in coconut peat and vermiculite substrates, revealing the distinct impacts of these substrates on species interconnectivity. The co-occurrence network visualizations (Figure 8) demonstrate that the networks in coconut peat-containing groups (233 nodes, 527 edges) are more complex compared to those in vermiculite-containing groups, which have fewer nodes (46) and edges (46). The coconut peat networks showed 90.13% positive and 9.87% negative correlations, while vermiculite networks had 97.83% positive and 2.17% negative correlations. In both substrates, Proteobacteria played a pivotal role in microbial network interactions (coconut peat: 48.07%, vermiculite: 63.04%). Notably, in the coconut peat group, 75 edges represented interactions between bacteria and fungi, accounting for 14.2% of the network; in the vermiculite group, there were 6 such edges, accounting for 13.0%. The presence of coconut peat led to more frequent microbial intercommunication and also increased the negative interactions between bacteria and fungi.

### 3.5. Influence of Substrate Physicochemical Properties on Microbial Communities

The experimental data and results analysis related to environmental factors are detailed in Appendix A.

Variance partition analysis (VPA) was conducted to ascertain the contributions of different environmental factors to community variation. The results indicated that in both bacterial and fungal communities, physical factors (such as WSN, SP, AFP, WHP, EC) and chemical factors (including TOC, TN, TP, TK, AP, AK, pH) together accounted for a substantial portion of community variation (44% for bacteria and 61% for fungi). Specifically, chemical factors were responsible for 39% and 25% of the variation in bacterial and fungal communities, respectively, while individual physical factors contributed relatively minor proportions (7% for bacteria and 6% for fungi) (Figure S5).

**Figure 9:**
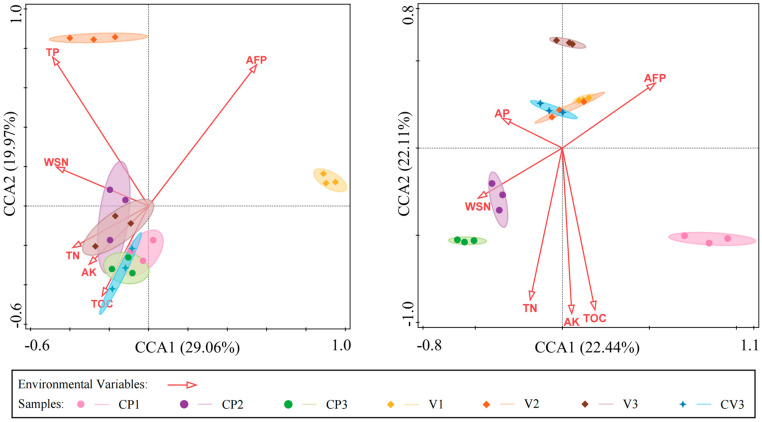
The canonical correspondence analysis (CCA) demonstrates the influence of environmental factors on microbial community composition (at the genus level) under various treatments, showcasing only factors with *p* < 0.05. The left panel presents the results of bacterial genus CCA analysis, while the right panel displays the fungal genus CCA analysis.

Canonical correspondence analysis (CCA) further validated the significant influence of environmental factors on microbial communities at the genus level. In the bacterial community (Figure 9 left), factors such as AFP, TP, WSN, TN, AK, and TOC showed significant correlations, together explaining a major part of the variation (Table S6a). For the fungal community (Figure 9 right), AFP, AP, WSN, TN, AK, and TOC were significantly correlated, collectively accounting for a large proportion of the variation (Table S6b). Constrained analysis revealed that the first two axes accounted for 49.03% and 42.55% of the variation in bacterial and fungal communities, respectively. Notably, the bacterial community exhibited two distinct groupings based on environmental factor influences: CP1~CP3, V3, and CV3 demonstrated similar responses with significant positive correlations to TN, AK, TOC, and a negative correlation to AFP. Conversely, V1 and V2 showed opposing characteristics. In the fungal community, the primary factors affecting CP2 and CP3 were WSN and AFP, with a significant positive correlation to WSN and a negative correlation to AFP. V13, and CV3 exhibited significant positive correlations with AP and AFP, and negative correlations with TN, AK, and TOC. The fungal community in CP1 responded differently to environmental factors compared to the other groups.

**Figure 10:**
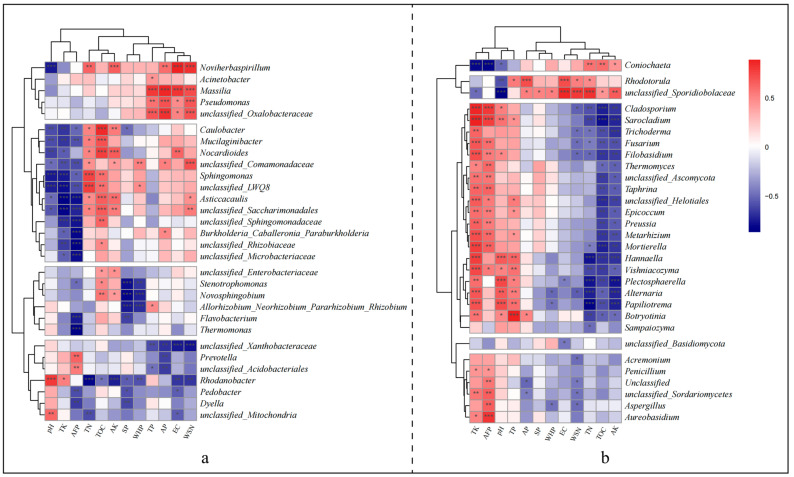
This heatmap illustrates the correlations between environmental factors and species based on Spearman’s analysis. Clustering, based on Euclidean distance, was performed separately for rows and columns, with row clustering grouping similar fungal genera. Each cluster represents a set of genera that share closer correlations with specific environmental factors. (**a**) Interrelationships between the top 30 bacterial genera in relative abundance and environmental factors; (**b**) Interrelationships between the top 30 fungal genera in relative abundance and environmental factors. Red indicates a positive correlation, while blue signifies a negative correlation. Significance was analysed using the BH-corrected *p*-value: “***” denotes *p* < 0.001, “**” *p* < 0.01, “*” *p* < 0.05, and unmarked correlations are not significant.

Spearman’s correlation heatmap further elucidates the distinct responses of bacterial and fungal genera to environmental factors. For bacteria, four clusters are identifiable based on their responses to environmental factors (Figure 10a). Cluster 1 shows a significant positive correlation with TP, AP, EC, and WSN; cluster 2 primarily exhibits a significant positive correlation with TOC, TN, and AK, but a significant negative correlation with pH, TK, AFP; cluster 3 predominantly displays a significant negative correlation with AFP, SP, WHP; and cluster 4 mainly shows a strong negative correlation with TN, AK, WHP, AP, EC, and WSN. Fungal genera are divided into five clusters (Figure 10b), with Cluster 1’s Coniochaeta significantly negatively correlating with TK, AFP, pH and significantly positively with TN, TOC, AK; Cluster 2 showing significant positive correlations with AP, EC, WSN, TN, and a significant negative correlation with pH; Cluster 3 primarily presenting a significant positive correlation with TK, AFP, pH, TP and a significant negative correlation with TN, TOC, AK; Cluster 4 solely exhibiting a significant negative correlation with EC; Cluster 5 mostly having significant positive correlations with TK and AFP, but significant negative correlations with AP, WHP, WSN, etc.

## 4. Discussion

### 4.1. Differential Microbial Diversity in Coconut Peat and Vermiculite

Our study reveals distinct microbial diversity patterns in coconut peat and vermiculite, following the addition of base fertilizer and three days of composting, as well as after fifty days of potato cultivation. In coconut peat, we observed a progressive increase in bacterial community diversity, richness, and evenness. Conversely, vermiculite experienced a significant decrease in both bacterial and fungal community diversity. The bacterial community in vermiculite showed lower evenness compared to coconut peat, whereas the fungal community demonstrated higher evenness than in coconut peat.

#### 4.1.1. Differential Bacterial Diversity in Coconut Peat and Vermiculite

Initially, both substrates displayed high bacterial diversity and richness, with the vermiculite group (V1) exhibiting the greatest diversity. Ana S.R. et al.’s [184] research on cave mineral layers, which are nutrient-deficient, also found higher bacterial diversity compared to typical soil. This suggests that microbial communities in extreme environments, such as rock surfaces, often display enhanced synergism, allowing them to utilize substances from minerals and the atmosphere [22]. Thus, the observed phenomenon may relate to the greater ecological niche specialization and interdependence among bacterial groups in nutrient-poor environments, crucial for nutrient cycling. The lower PD_whole_tree index [185] in the V1 group further suggests closer evolutionary relationships within the microbial community on its surface. In cultivation systems, the microbial functions and evolutionary traits introduced by vermiculite are more concentrated, whereas coconut peat introduces a broader array of traits.

Three days post-composting, we noted significant reductions in bacterial diversity and richness in both substrates, with the remaining bacterial communities becoming more closely related. Some studies indicate that in artificially created, nitrogen-rich environments, intense competition for scarce resources can cause widespread microbial death and a decrease in soil microbial diversity [186]. Additionally, the sudden influx of nutrients can prompt rapid colonization by dominant or specialized functional bacteria. Moreover, the elevated temperatures from fermentation may eliminate heat-sensitive microbes. However, this reduction in diversity was more pronounced in vermiculite. This mirrors Anna A.G. ‘s [22] findings that microbes long exposed to extreme environments are particularly susceptible to shifts when growth conditions momentarily improve. We hypothesize that the sharp decline in bacterial diversity in vermiculite results from disrupting the synergistic equilibrium between mineral-decomposing bacteria and a minority of organic nutrient-dependent bacteria, triggered by the sudden nutrient increase. This shift leads to the proliferation of bacteria tolerant to various stresses, significantly reducing the ecological niches for mineral-decomposing bacteria and resulting in their disappearance.

Upon examining the rhizosphere substrates of potatoes following fifty days of cultivation, divergent trends were noted between coconut peat and vermiculite. Coconut peat substrates exhibited a marked increase in microbial diversity and richness, whereas vermiculite substrates displayed a decline. Given that cultivation was performed under identical external conditions, using the same seed stock, and with rigorous controls on microbial introduction during irrigation, these contrasting outcomes are attributable to the inherent properties of the substrates themselves or the differences in nutrient distribution they induce, coupled with plant secretions. The variation in microbial communities associated with nutrient decomposition and utilization introduced by each substrate suggests a lesser degree of microbial population differentiation in vermiculite than in coconut peat. This disparity becomes more pronounced following the enrichment of microbes by plant root secretions.

#### 4.1.2. Differential Fungal Diversity in Coconut Peat and Vermiculite

Across all groups, fungal community diversity was found to be highest in V1 and V2, yet no significant differences in richness or phylogenetic diversity were observed. This parallels Y. Colin et al.’s [187] findings, where mineral surfaces hosted a greater diversity of fungi compared to bulk soil. Typically, due to limited moisture and organic matter, fungi on mineral surfaces tend not to produce extensive hyphae but rather exist as independent epiphytic or microcolonial fungi [188]. Consequently, in environments where large fungal communities do not dominate ecological niches, a wider range of fungal species can colonize vermiculite surfaces. The elevated fungal diversity in vermiculite likely due to the fact that it provides colonisation sites for a large number of microcolonising fungi and reflects intricate microbial interactions on its surface. Notably, the timing of significant fungal diversity changes varies between coconut peat and vermiculite: in coconut peat, notable fluctuations occur after three days of combined fermentation, whereas in vermiculite, these shifts emerge after fifty days of potato cultivation. Despite the absence of significant richness alterations across all groups, it is posited that indigenous fungi in vermiculite maintain functions analogous to those in coconut peat. Fluctuations in species evenness could result from a variety of factors, including changes in medium and bacterial community dynamics.

In conclusion, organic coconut peat and inorganic vermiculite differentially impact microbial diversity and richness throughout the cultivation cycle, critically influencing the overall health-promoting capacity of the planting environment. The persistent decrease in bacterial diversity within vermiculite substrates may signal a less favorable rhizospheric environment for plants, potentially heralding an uptick in soilborne diseases [189]. Furthermore, when fungal richness remains elevated, it suggests a disruption in the optimal bacterial-fungal balance within vermiculite systems, skewing the ecosystem towards a fungal dominance detrimental to plant health [190].

### 4.2. Microbial Community Dynamics Associated with Substrates

Our analysis identified distinct microbial community succession patterns in coconut peat and vermiculite, following base fertilizer addition and three days of composting, as well as after fifty days of potato cultivation. In coconut peat, the bacterial community showcases a predominance of specific dominant phyla and genera, whereas the fungal community is governed by a limited range of phyla and genera. In contrast, bacterial taxa in vermiculite are more broadly distributed, with a fungal community composition that is more evenly dispersed. These findings, across three sampling intervals, indicate that microbial interactions in coconut peat may more closely align with the balance required for plant health, in contrast to vermiculite, where the dynamics between beneficial and pathogenic microbes often surpass the equilibrium necessary for optimal plant growth.

#### 4.2.1. Dynamics of Bacterial Communities Associated with Substrates

Across all groups except for single vermiculite (V1), *Proteobacteria* emerged as the dominant bacterial phylum, succeeded by *Bacteroidota* and *Actinobacteriota*. In contrast, V1 demonstrated a more even distribution among phyla, suggesting that outside of V1, bacterial community interrelations are primarily influenced by *Proteobacteria*. Conversely, the microbial equilibrium in vermiculite reflects the synergistic interplay among various dominant phyla, promoting a more stable community overall [24]. At the genus level, V1 showcases low species abundance yet harbors complex diversity. Despite potential functional similarities, subtle genomic variations among species on vermiculite surfaces may enhance their collaborative survival in harsh environments [22]. Following base fertilizer application and three days of fermentation, coconut peat’s microbial stability increases, with notable shifts in *Bacteroidota* and *Actinobacteriota* proportions, yet overall phyla dominance remains largely intact. Vermiculite, however, undergoes significant phylum evenness adjustments, with Proteobacteria representing over 85% of the community. This shift may disrupt community stability due to inadequate functional diversity for rapid microbial growth in nutrient-rich conditions. At the fifty-day mark with plant integration, both substrates converge towards a similar microbial profile at the phylum level, primarily dominated by Proteobacteria and Bacteroidota, influenced heavily by the plant rhizosphere. However, minor differences in low-abundance phyla reflect the substrate’s ongoing impact on community dynamics.

Analysis of the top 15 bacterial genera by relative abundance highlighted significant genus-level differences between substrates. V1′s minimal representation among these genera underscores the complexity of microbial networks on vermiculite’s surface. Post 3d-basal fertilizer and composting, both substrates predominantly featured *Massilia*, critical for compound decomposition and utilization. Vermiculite exhibited a pronounced increase in *Massilia* and *Pseudomonas*, indicating their role in destabilizing the microbial community due to vermiculite’s lower species richness. Fifty days into potato cultivation, species evenness peaked in CV3, with CP3 and V3 following suit. CP3 demonstrated broader bacterial diversity than V3, suggesting a cooperative microbial assembly in coconut peat versus a few dominant genera in vermiculite.

LEfSe analysis confirmed a strong correlation between substrate type and rhizospheric bacterial community traits. In coconut peat (CP3), enriched microorganisms associated with polysaccharide degradation and denitrification, such as *Verrucomicrobiota* [191] and *Caulobacterales* [192], suggest coconut peat’s high cellulose content fosters *Caulobacterales* proliferation, enhancing survival for other sugar-metabolizing microbes. *Methylophilaceae*, also enriched in CP3, known for denitrification [193,194], contribute to nitrate conversion and participate in dimethyl sulfide (DMS) and methylated compound cycling [195,196], likely benefiting from plant tissue decomposition [197]. This indicates coconut peat’s microbial community fosters an efficient carbon and nitrogen utilization environment for plants. In vermiculite (V3), enriched bacteria such as *Micrococcales*, *Flavobacterium*, and *Stenotrophomonas* hold significant plant rhizosphere associations and diverse functions [198,199,200]. Notably, *Micrococcales* [201] and *Pectobacterium* [202,203,204,205,206] exhibit strong plant pathogenicity, while *Stenotrophomonas* [199] offers antibacterial and plant growth-promoting activities.

#### 4.2.2. Dynamic Changes in Fungal Communities Associated with Substrates

*Ascomycota* and *Basidiomycota*, the predominant fungal phyla, are present in all groups, accounting for over 90% of the microbial communities [187,207,208,209]. Demonstrating strong adaptability, these phyla are widely distributed across various environments, such as soil, rocks, and organic matter, thus dominating microbial communities. The fungal community in coconut peat (CP1) primarily consists of *Ascomycota*, in contrast to the more diverse and evenly distributed fungal phyla in vermiculite (V1), likely due to the complex microbial associations on the mineral surface. The addition of basal fertilizer and a subsequent three-day fermentation process increased the relative abundance of *Basidiomycota* in both substrates. However, the fungal community’s stability in the vermiculite substrate (V2) was significantly higher than in coconut peat (CP2), with a more pronounced increase in *Basidiomycota* in the latter. Research by Luisa M.M. et al. [210] indicates that *Ascomycota* possess a broader spectrum of enzymes than *Basidiomycota*, enabling a wider utilization of organic compounds. Despite this, *Ascomycota* exhibit higher genetic variability within the same genus encoding the same enzymes. This suggests *Basidiomycota*’s more stable role in degrading certain recalcitrant substances, possibly explaining their significant increase in CP2. With plant integration into the system (at the 50-day mark), *Ascomycota* represented over 90% of the abundance in coconut peat, while *Basidiomycota* were more prevalent in vermiculite, suggesting a substantial presence of recalcitrant substances in the latter awaiting degradation. In CV3, the relative abundance of *Ascomycota* and *Basidiomycota* was more balanced, indicating a regulatory role of both substrates on these phyla.

At the genus level, significant variability exists in coconut peat across the three stages, with *Aspergillus*, *Rhodotorula*, and *Coniochaeta* predominating in CP1, CP2, and CP3, respectively. In contrast, the fungal genera in the vermiculite substrate maintained a relatively stable structure during the first two stages, with *Cladosporium*, *Fusarium*, *Alternaria*, *Mortierella*, and *Filobasidium* being the top five genera in relative abundance. However, significant shifts in fungal genera distribution in the vermicite substrate occurred after 50 days, with *Papiliotrema* emerging as dominant in V3. Compared to CP3 and V3, the evenness of fungal genera composition in CV3 was significantly higher, suggesting the regulatory role of coconut peat and vermiculite’s distinct characteristics on the composition of fungal genera in the potato rhizosphere.

The LEfSe analysis reveals that biomarker types are closely related to the nutritional and environmental conditions provided by coconut peat and vermiculite. Compared to coconut peat, vermiculite’s enriched fungi tend to display antagonistic or competitive relationships, with an enrichment of both pathogenic fungi and those exhibiting antipathogenic activities. In the coconut peat substrate, *Calosphaeriaceae* [211,212,213] and *Aspergillus* [214,215] are predominant in CP1, whereas *Rhodotorula* [216] and *unclassified_sporidiobolaceae* (both from *Sporidiobolaceae*) dominate in CP2, and *Coniochaeta* [217,218] in CP3. These microorganisms, adept at degrading woody fibers and commonly found on bark and decaying wood, utilize complex plant polymers as their primary food source. *Rhodotorula* and *Coniochaeta*, enriched in CP2 and CP3, are identified as potential plant growth-promoting fungi, known for synthesizing antipathogenic compounds, plant hormones, iron acquisition, and phosphate breakdown [219,220]. In the vermiculite substrate, *Cladosporium*, *Trichocomaceae*, *Fusarium*, *Mortierella*, and *Agaricales*, enriched in V1, exhibit strong adaptability to harsh conditions, capable of surviving in environments with low nutrient availability, limited water, and extreme temperatures [221,222,223]. This resilience aligns with the dry, nutrient-poor conditions of vermiculite. *Trichocomaceae*, *Mortierella*, and *Fusarium*, in particular, have notable metabolic and redox capabilities essential for nutrient utilization and microbial community stabilization on vermiculite [224,225]. Enriched fungi in V2 of vermiculite include species tolerant to extreme conditions (low nutrients, extreme temperatures) and exhibit complex host-related traits [226,227,228,229,230], such as *Fusicolla*’s ability to degrade recalcitrant substances, and *Didymellaceae*’s antifungal and potential pathogenic activities against most plants [231,232]. In V3, *Botryotinia* [233,234], a common plant pathogen, is enriched alongside fungi (*Hannaella* [235] and *Papiliotrema* [236]) with capabilities to enhance plant resistance, synthesize defensive substances, and degrade pathogen cell walls, indicating potential mutual competition and inhibition among vermiculite’s enriched fungi.

In conclusion, distinct patterns of microbial community changes are evident between the two substrates. In vermiculite, biomarkers reflect more interactions with plants, displaying competitive and antagonistic tendencies, while coconut peat’s biomarkers are linked to efficient nutrient decomposition and utilization. Additionally, the relationship between fungi and substrate properties is significant, with vermiculite’s fungal biomarkers often found in extreme environments, suggesting these species play a crucial regulatory role in the fungal community.

### 4.3. Alterations in Microbial Community Functions in Substrates

#### 4.3.1. Variations in Bacterial Community Functions in Substrates

A statistical analysis was conducted on 11 functions of plant growth-promoting rhizobacteria (PGPR) enriched in each group, including “siderophore production,” “nitrogen fixation,” “phosphorus solubilization,” among others. This analysis revealed that the substrate significantly influences the enrichment of PGPRs with specific functions. Initially, coconut peat was found to inherently support a higher abundance of bacteria with PGPR functions, corroborating findings by A. SharaS et al. [18]. Post-composting, a decline in these PGPR levels in coconut peat was observed, suggesting that competitive nutrient acquisition in environments with high temperatures or nutrients may limit their prevalence. Conversely, vermiculite showed a notable increase in PGPR abundance post-composting, likely due to the enhanced utilization of nutrients by initially less abundant organic nutrient-type microorganisms, facilitating their population growth. After 50 days of potato cultivation, a resurgence of PGPR abundance was noted in the coconut peat’s potato rhizosphere, whereas a significant reduction was observed in vermiculite. This pattern, as suggested by preceding LEfSe analysis, indicates potentially higher competition and antagonism among bacteria in vermiculite in the presence of plants, affecting the enrichment of function-specific PGPR. Comparative analysis of rhizosphere samples from three groups (CP3, V3, CV3) indicated uniform levels of characteristic PGPR across them. This suggests that both substrates, including their mixture, successfully introduce diverse beneficial microorganisms into the plant rhizosphere, retaining certain characteristic PGPR from each substrate, unaffected by the blend of coconut peat and vermiculite.

Functional analysis through FAPROTAX identified “chemoheterotrophy” and “aerobic chemoheterotrophy” as the dominant bacterial functions across all groups. At the second sampling stage (CP2, V2), significant increases in both “aerobic chemoheterotrophy” and “ureolysis” were observed in coconut peat and vermiculite, correlating with basal fertilizer addition. This stage marked extensive nutrient decomposition by bacteria, promoting the proliferation of dominant species.

In general, an increase in organic matter, nitrogen, phosphorus, and other elements enhances microbial growth and metabolic activity, enriching associated metabolic pathways. From the second to the third stage, a decrease in “ureolysis” and “aromatic compound degradation” functions was noted, likely due to nutrient depletion. However, pathways such as carbon metabolism, oxidative phosphorylation, and amino acid biosynthesis remained enriched in both substrates post-planting (CP3, V3), despite significant nutrient reduction. This suggests that specific microorganisms with unique functions facilitate nutrient redistribution and utilization under short-term planting conditions, compensating for the limited metabolic activities of others and maintaining ecosystem stability. Furthermore, an increase in antibiotic biosynthesis could signify enhanced microbial communication or competition, pivotal for the structure and stability of communities in soilless cultivation conditions.

After 50 days post potato cultivation, we noted distinct trends in the functional transformations within the bacterial communities of two different substrates. In the substrate CV3, both “Chemoheterotrophy” and “aerobic chemoheterotrophy” exhibited a rising trend, contrary to their decline observed in substrate V3. In the latter, bacterial functions veered towards “nitrate reduction” and “nitrate respiration,” indicative of denitrification processes [237,238]. Literature indicates that sulfur and nitrifying bacteria are often found on weathered rocks’ surfaces [27,239,240], suggesting that vermiculite provides a conducive environment for communities engaged in nitrate reduction. When comparing coconut peat (CP3) with vermiculite (V3), the rhizosphere of the latter showed an enrichment in pathways related to pyrimidine and purine metabolism. This enrichment, notably absent in vermiculite’s natural composition, arises post-cultivation, likely due to the influence of plant root exudates [241]. It suggests that plants might release nucleotides to either fend off pathogenic microbes or to foster beneficial interactions within the rhizosphere. Such biochemical exchanges could shift the competitive landscape and ecological equilibrium among rhizospheric microorganisms, potentially elucidating the observed decrease in PGPR enrichment during later stages of cultivation in vermiculite.

#### 4.3.2. Variations in Fungal Community Functionality across Substrates

Previous studies highlight the presence of potential human or animal pathogens in compost, a concern seemingly independent of the compost’s materials or preparation methods [242]. This observation aligns with the noted increase in the abundance of animal endosymbionts, pathogens, and endophytic fungi in substrates CP2 and V2. Throughout the composting process, both substrates exhibited a decrease in the prevalence of undefined saprotrophs. However, the abundance of plant pathogens diminished in CP2 but escalated in V2.

The contrast in fungal functional composition between the coconut peat and vermiculite substrates was most pronounced in the third stage, especially regarding plant pathogens, undefined saprotrophs, and wood saprotrophs. In CP3, there was a noticeable increase in the relative abundance of undefined and wood saprotrophs within the plant rhizosphere, alongside a significant decline in plant pathogens. This pattern mirrors findings in the *Artemisia annua* rhizosphere [243]. Saprotrophic fungi, key in decomposing root exudates, dead root tissues, and leaf litter, are essential for nutrient recycling and environmental health [244,245,246]. The marked rise in saprophytic fungi in CP3 stems from these plant-derived materials. Conversely, V3 saw a reduction in saprophytic fungi, supplanted by an increase in plant pathogens. This shift may be attributed to the saprotrophic fungi’s struggle to thrive due to vermiculite’s extremely low cellulose content, leaving plant pathogens to dominate this niche by default.

Understanding the correlation networks between fungi and bacteria is pivotal in discerning the nature of their interactions and their environmental impact. Our observations indicate that the microbial correlation network in coconut peat substrates is more intricate, featuring more interactions between bacteria and fungi, including significant negative correlations. Prior studies suggest that bacterial-fungal communication often leans towards mutual competition and inhibition, as beneficial bacteria can produce antibiotics to counter pathogens and competitively inhibit the growth of pathogenic bacteria [247]. Moreover, reduced complexity in bacterial interactions has been implicated in continuous cropping challenges [248]. In this study, the presence of beneficial saprophytic fungi and increased negative inter-kingdom correlations between bacteria and fungi in coconut peat substrates seem to facilitate the shift towards a “bacterial-type” community. This shift potentially enhances the plant’s resistance to pathogenic fungal attacks [249].

### 4.4. Primary Environmental Factors Influencing Microbial Community Composition

CCA and correlation heatmap analyses underscore the substantial influence of substrate physicochemical factors on microbial community structure and diversity. In soilless cultivation, pivotal factors shaping bacterial and fungal community dynamics include AFP, TOC, TN, AK, and WSN. Contrary to expectations, the relationship between EC and microbial composition was not significant, suggesting that the elevated salinity in coconut peat does not primarily drive microbial variations. The key determinants for microbial community composition are essential nutrients and substrate aeration capacity. Our findings also indicate a differential response to phosphorus between bacteria and fungi; bacteria have a significant correlation with TP, whereas fungi show a correlation with AP, likely reflecting some bacteria’s ability to solubilize phosphorus. Correlation analysis reveals that dominant bacterial genera are negatively associated with AFP and positively with WSN, TN, AK, and TOC, while dominant fungal genera display the opposite pattern. This suggests that nutrient decomposition and utilization are predominantly bacterial processes. The presence of fungi negatively correlated with these nutrient parameters may stem from bacteria’s superior reproductive and competitive capabilities, which enable them to rapidly colonize ecological niches following nutrient influxes, thus suppressing fungal community development.

## 5. Conclusions

In the realm of long-term soilless cultivation, substantial evidence indicates that plants grown in either purely organic or hybrid organic/inorganic substrates frequently demonstrate superior growth characteristics compared to those in purely inorganic mediums. Despite this, the literature has sparingly addressed the influence of substrate-induced alterations in microbial communities on plant growth. This study contributes insights into the potential effects of varied microbial environments, engendered by substrate properties, on plant production within soilless systems. First, coconut peat and vermiculite introduce distinct microbial populations into the cultivation environment. Coconut peat enriches the environment with a plethora of organic nutrient-utilizing microorganisms, adept at breaking down complex organic matter. In contrast, vermiculite harbors a dual microbial community of mineral and organic nutrient-utilizing microorganisms, exhibiting intricate synergistic interactions. Second, vermiculite’s diverse and stable indigenous microbial community is susceptible to destabilization upon the introduction of base fertilizers. The abrupt nutrient surge can foster dominance by more aggressive microbial species, escalating competition and aggression within the community and undermining stability at the plant rhizosphere (a critical factor in the observed microbial community shifts). Third, while plant growth can attract more plant growth-promoting rhizobacteria (PGPR), the primary microbial groups’ composition and function remain influenced by the native microbial community within the substrate. Fourth, the nutrient conditions provided by water-soluble nitrogen (WSN), total nitrogen (TN), available potassium (AK), and total organic carbon (TOC) favor bacterial proliferation in substrates, whereas available phosphorus (AFP) benefits fungal growth, likely due to bacteria’s rapid nutrient utilization and niche occupation capabilities. In conclusion, from a microbial standpoint, the relatively homogenous microbial functional structure and nutritional profile in coconut peat play a significant role in its beneficial impact on plant growth. Conversely, the complex microbial interactions in vermiculite are ill-suited for the abrupt nutrient changes encountered during cultivation, suggesting a need for meticulous and long-term nutrient environment adjustments (including WSN, TN, AK, TOC, etc.) to harmonize vermiculite’s surface microbial communities for optimal plant cultivation conditions.

## Data Availability

Relevant microbial sequence files and metadata from this study are available in the NCBI BioProject database under accession number PRJNA999698 (https://www.ncbi.nlm.nih.gov/sra/PRJNA999698, accessed on 28 July 2023).

## Supplementary Materials

The following supporting information can be downloaded at: https://www.mdpi.com/article/10.3390/microorganisms12030584/s1, Supplementary figures and tables referenced in the main text are available in Supplementary Data S1, while the original OTUs data and code run results can be found in Supplementary Data S2.

## Appendix A. Changes in Physical and Chemical Properties of Cultivation Substrates

This appendix highlights the changes in the physical and chemical properties of the samples, focusing on 12 key indicators such as pH, EC, TOC, TN, and more (Table A1). The results indicate that, compared to pure coconut peat (CP1), pure vermiculite (V1) had a more neutral pH, significantly lower EC value, and the lowest nutrient element contents (C, N, P, AK) among all groups, but exhibited higher TK and porosity than CP1. The addition of base fertilizer to both coconut peat and vermiculite led to reduced pH and TK, alongside increases in EC, TN, TP, AP, and WSN. Post-fertilization, TOC and AK in coconut peat decreased, contrary to the increase observed in vermiculite, potentially due to coconut peat’s high cellulose, lignin, and salt content. Additionally, SP and WHP significantly increased in coconut peat but remained relatively unchanged in vermiculite. Following a 50-day planting period, both substrates experienced a rebound in pH and a decline in EC. The indicators for TN, TP, TK, AP, WSN, and porosity reduced variably. However, compared to CP2, the TOC content in CP3 slightly decreased and the AK content increased, while in vermiculite (V2 to V3), TOC percentage increased and AK decreased. Furthermore, as the proportion of coconut peat increased and that of vermiculite decreased, pH consistently dropped towards weak acidity, and TOC, TN, SP, and WHP progressively rose, while TP, AP, and AFP decreased in the V3, CV3, CP3 rhizosphere samples. It was also noted that potato planting tended to revert the samples’ physical and chemical properties to their pre-fertilization state.

**Table A1:** Physical and Chemical Properties of Soil Under Different Treatments.

	CP1	CP2	CP3	V1	V2	V3	CV3
pH	6.15 ± 0.01 d	5.96 ± 0.01 e	6.54 ± 0.10 c	7.09 ± 0.02 a	6.60 ± 0.01 c	7.07 ± 0.02 a	6.94 ± 0.02 b
EC (uS/cm)	723.33 ± 5.81 c	1156 ± 5.13 a	318.33 ± 2.19 e	57.1 ± 0.66 g	1056 ± 3.06 b	560.33 ± 3.48 d	208.33 ± 1.76 f
TOC (%)	45.81 ± 1.31 a	31.10 ± 0.54 b	30.48 ± 0.70 b	0.22 ± 0.01 f	0.32 ± 0.01 e	0.45 ± 0.01 d	4.91 ± 0.01 c
TN (g/kg)	8.61 ± 0.01 b	11.34 ± 0.37 a	8.55 ± 0.14 b	2.83 ± 0.03 e	5.21 ± 0.08 c	2.40 ± 0.04 f	4.89 ± 0.10 d
TP (%)	0.13 ± 0.01 d	0.23 ± 0.01 c	0.07 ± 0.00 e	0.11 ± 0.01 d	0.55 ± 0.02 a	0.34 ± 0.01 b	0.25 ± 0.01 c
TK (%)	0.76 ± 0.01 d	0.66 ± 0.02 e	0.63 ± 0.02 e	3.22 ± 0.05 a	2.80 ± 0.11 b	2.77 ± 0.14 b	2.24 ± 0.09 c
AP (mg/kg)	90.83 ± 7.25 d	1215.73 ± 96.30 a	156.90 ± 8.88 c	4.63 ± 0.13 e	1129.67 ± 56.82 a	547.67 ± 17.89 b	507.63 ± 10.54 b
AK (mg/kg)	10750.67 ± 357.45 a	8168.33 ± 145.73 c	8998.33 ± 349.96 b	111.67 ± 1.45 g	2219.33 ± 4.33 d	313.33 ± 8.65 e	208.67 ± 0.33 f
WSN (mg/kg)	283.73 ± 2.22 e	2038.13 ± 16.65 a	677.67 ± 3.05 c	10.53 ± 0.30 g	886.23 ± 7.38 b	520.93 ± 3.35 d	158.37 ± 2.17 f
SP (%)	64.80 ± 0.76 c	72.83 ± 0.15 a	70.17 ± 0.24 b	72.27 ± 0.15 ab	72.00 ± 1.50 ab	64.90 ± 0.81 c	65.30 ± 0.59 c
AFP (%)	17.73 ± 0.12 b	16.87 ± 0.15 c	15.47 ± 0.09 e	19.20 ± 0.06 a	18.77 ± 0.09 a	17.07 ± 0.23 c	16.30 ± 0.23 d
WHP (%)	47.03 ± 0.65 c	56.00 ± 0.06 a	54.73 ± 0.32 ab	53.07 ± 0.12 b	53.23 ± 1.41 b	47.90 ± 0.64 c	49.00 ± 0.36 c

The data are mean ± standard error (SE, *n* = 3). The LSD test was used to judge significant differences between groups. The log was taken for the original data that did not satisfy the normal distribution and homogeneity test, and different lowercase letters indicate statistically significant differences at *p* < 0.05.
