# Supplementary material for: Exploring the Impact of Coconut Peat and Vermiculite on the Rhizosphere Microbiome of Pre-Basic Seed Potatoes under Soilless Cultivation Conditions
Source: Microorganisms. 2024 Mar 14;12(3):584. doi: 10.3390/microorganisms12030584 (PMC10974569; doi:10.3390/microorganisms12030584)

**Supplementary Figures**

**Figure S1.** Rarefaction curves of sequencing results for all samples. Different colours correspond to different samples. **a.** Bacteria; **b.** Fungi.


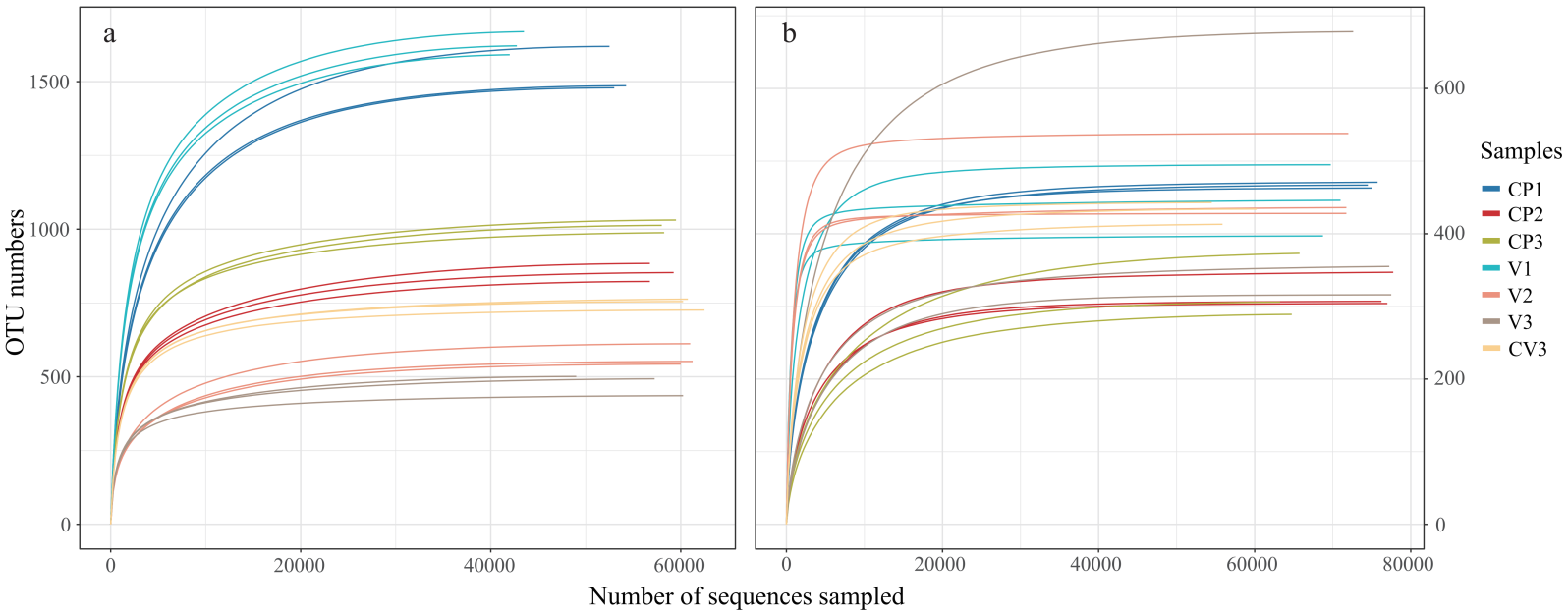


**Figure S2.** Non-metric multidimensional scaling (NMDS) showing the diversity of microbial

communities under different treatments, *P*<0.001. **a.** Bacteria; **b.** Fungi.


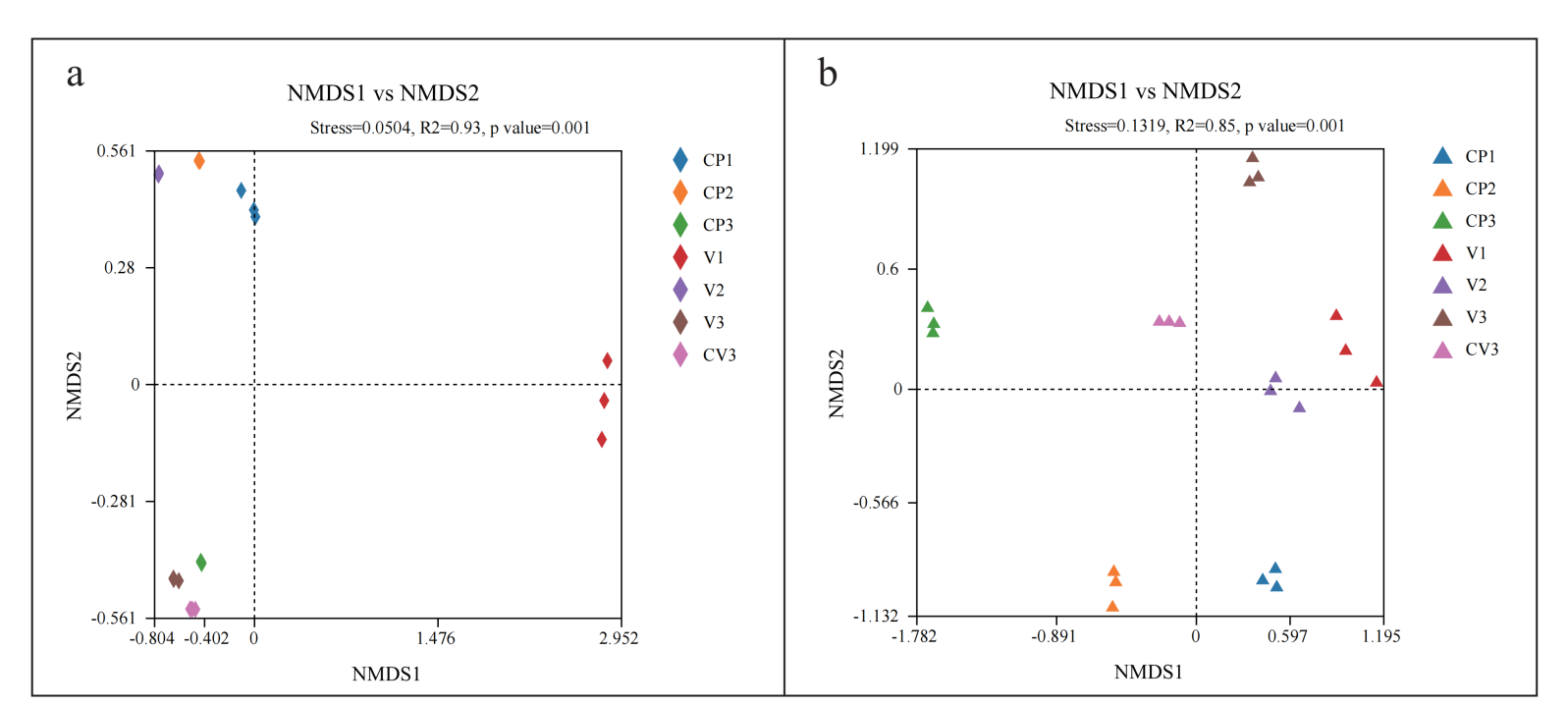


**Figure S3.** Shared and unique genus between different groups. **a.** Bacteria; **b.** Fungi.


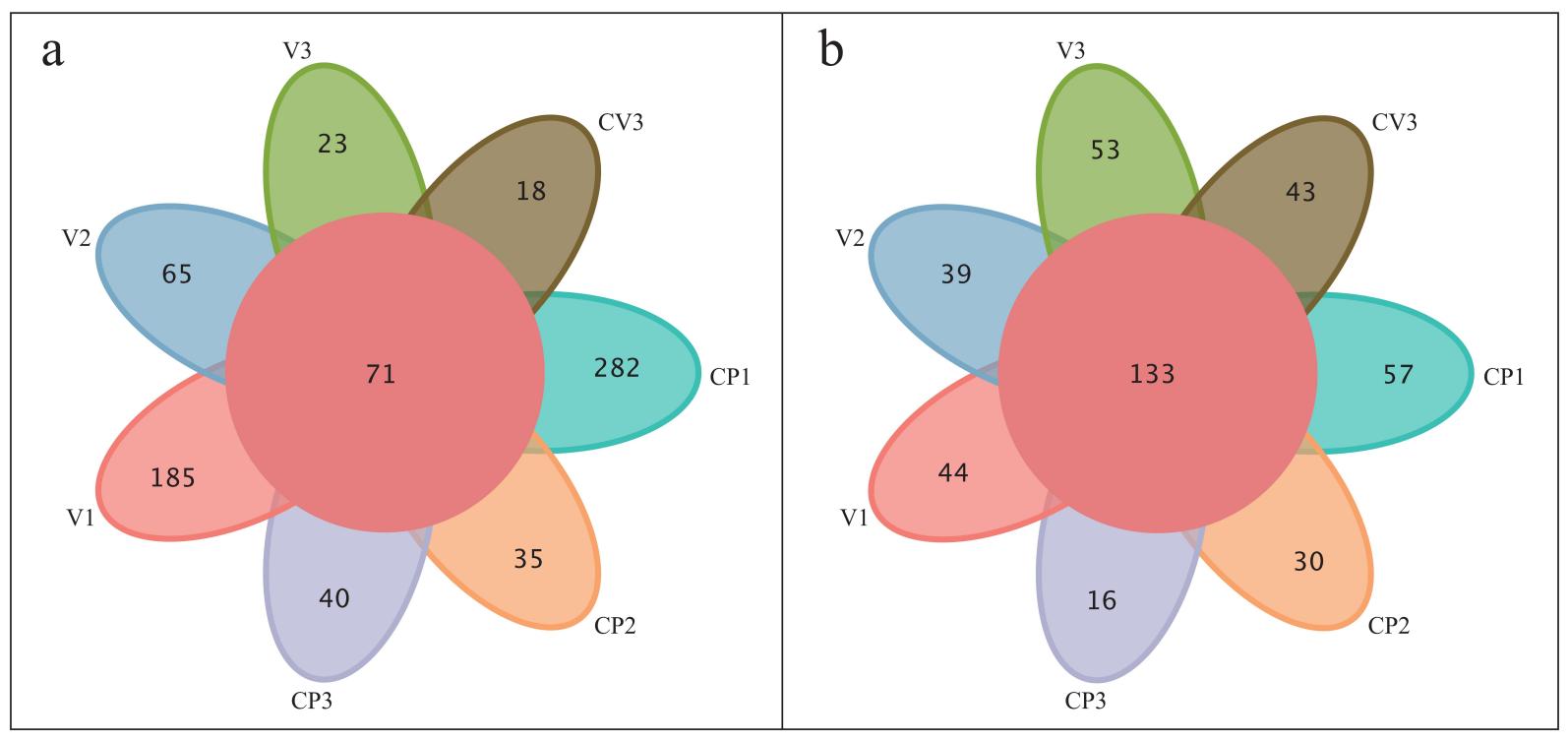


**Figure S4.** The LEfSe analysis results of fungi show taxonomic groups with statistical differences between the seven groups from phylum to genus (the non-parametric Kruskal-wallis rank sum test is employed to analyze microbial populations exhibiting significant differences among multiple groups. Set LDA＞4.0). The left image is an evolutionary branch diagram. The circles from inside to outside represent classification levels from phylum to genus, each small circle at different classification levels represents a category at that taxonomic level, and the size of the circle displays its relative abundance. The circles with corresponding colors in the figure represent the biomarker of that treatment, and yellow circles represent categories with no significant differences under corresponding LDA values. The legend on the right corresponds to biomarkers at different taxonomic levels in the same color group. The right image is a bar chart of LDA value distribution. It displays the biomarkers in each group.


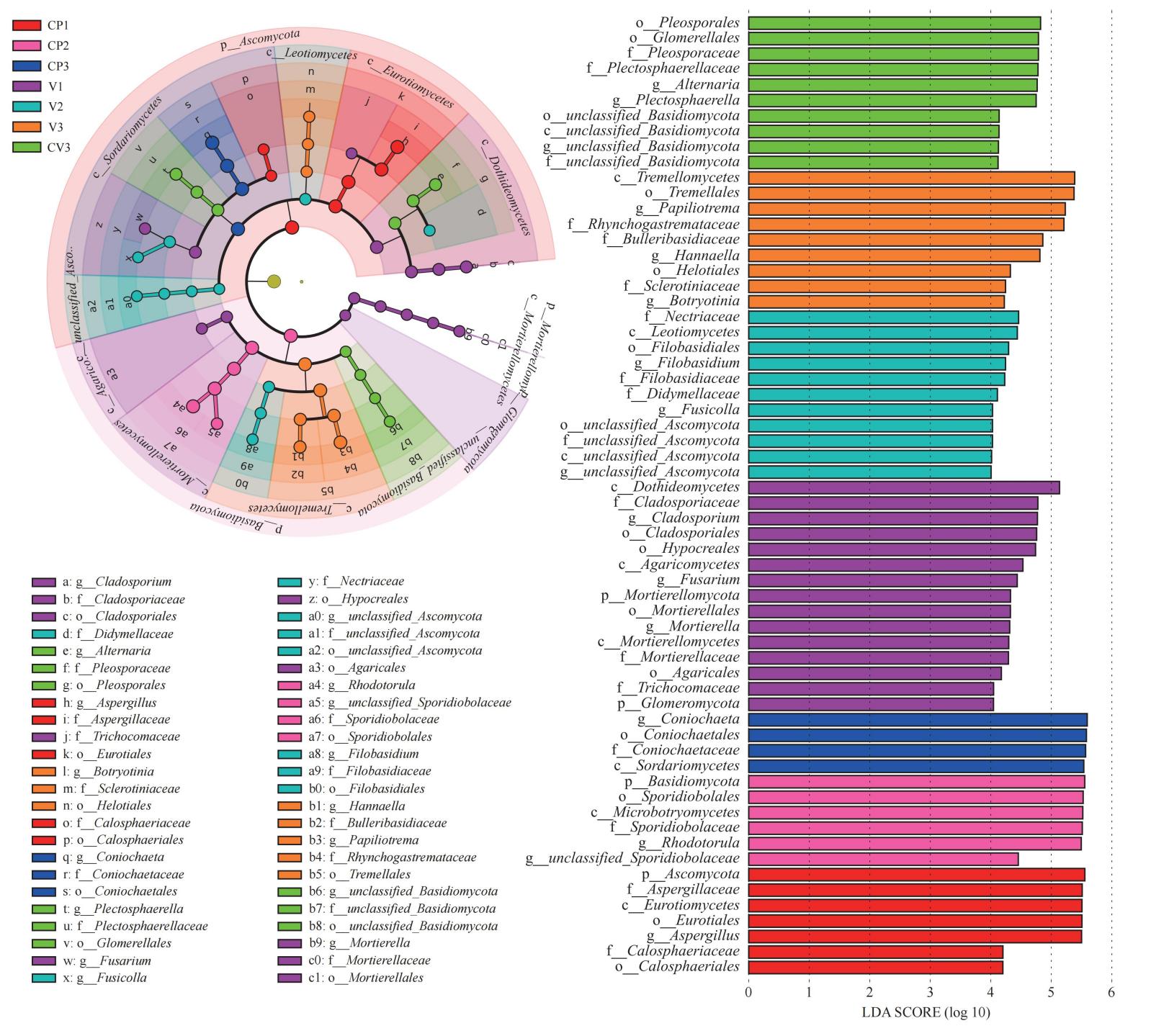


**Figure S5.** The VPA showing the independent and shared effects of physical and chemical factors on

community composition. The pink group represents chemical factors, including TOC, TN, TP, TK,

AP, AK, and pH. The blue group represents physical factors, including WSN, SP, AFP, WHP, and EC.

**a.** Bacteria; **b.** Fungi.


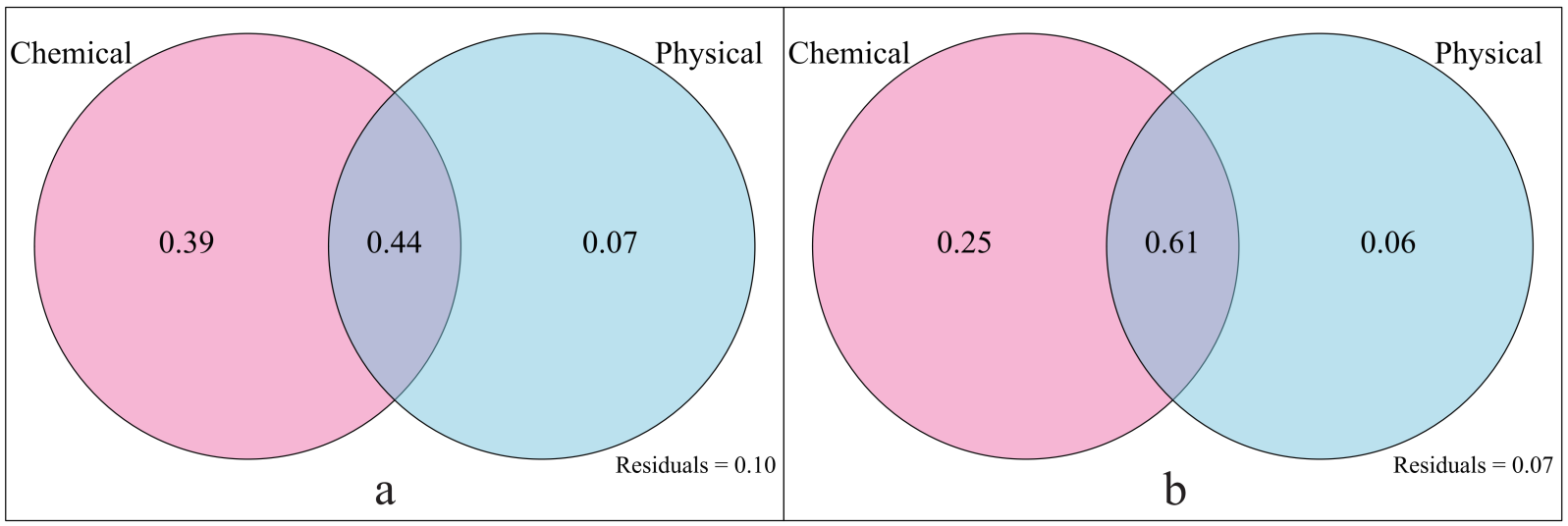

Supplement: Supplementary file 1 [file microorganisms-12-00584-s001.zip › Supplementary Files/Supplementary Data1/Supplementary Figures.docx]
